# Bringing art and science together to address climate change

**DOI:** 10.1007/s10584-025-03861-3

**Published:** 2025-03-06

**Authors:** Allyza R. Lustig, Allison R. Crimmins, Michael O. Snyder, Laura Tanner, Ian van Coller

**Affiliations:** 1 ICF, 1902 Reston Metro Plaza, Reston, VA 20190 USA; 2 National Oceanic and Atmospheric Administration, Washington, DC, USA; 3 S.I. Newhouse School of Public Communications, Syracuse University, 215 University Pl, Room 365, Syracuse, NY 13210 USA; 4 Florida Atlantic University, Boca Raton, FL USA; 5 Montana State University, Bozeman, MT USA

**Keywords:** Climate change, Visual art, Communication, Engagement, Assessment

## Abstract

Art x Climate was the first-ever gallery of visual art to be included in the National Climate Assessment. This letter outlines the purpose and process of Art x Climate and highlights three Art x Climate artists and their work. The letter concludes with lessons learned from this project: the need for cross-disciplinary respect among the arts and sciences, the wide range of themes and artworks centered around climate change, and the ability of art to facilitate new collaborations and bring more people into the climate change conversation.

## Introduction

Climate change occurs over such immense time and space, it challenges our ability to fully understand its scope. The arts expand people’s capacity to navigate, internalize, and place themselves within complex issues like climate change, fostering a personal connection to global crises and allowing people to connect across cultural and political lines (Li et al. 2023; Mah et al. 2024; Roosen et al. 2018; Kagan 2015). Artists hold the power to confront audiences with uncomfortable truths around risk, provoke discussion, and inspire individuals to take action and connect with community (Nurmis 2016; Ryan 2016; Bentz 2020). By way of example, in the United States, the 1968 Earthrise photograph radically changed social conceptions of humanity and the precariousness of this planet (Poole 2008). Fifty-five years later, when President Biden proclaimed October 2023 as National Arts and Humanities Month, he celebrated “all the artists and scholars who have dared to reveal the good, bad, and truth of our Nation, and, in the process, have strengthened the covenant that is our democracy” (The White House 2023a, b). The role of climate-related art to engage emotion, spark imagination, and prompt action has been invoked by major scientific institutions such as the Cultural Programs of the National Academy of Sciences and the American Geophysical Union’s 2022 and 2023 Art and Science Plenaries.

On this foundation, the U.S. Global Change Research Program (USGCRP) released its first-ever public call for visual art to be included in the Fifth National Climate Assessment (NCA5; USGCRP 2023). This call, named “Art x Climate,” expands on USGCRP’s mandate to “assist the Nation and the world to understand, assess, predict, and respond to human-induced and natural processes of global change” (Public Law 101–606: Global Change Research Act of 1990). Art x Climate sought to harness the power of art to make the issue of climate change more resonant with people across the country and to cultivate a more nuanced understanding of lived experiences.

This letter outlines the process behind the NCA5 Art x Climate gallery, with the hope that lessons learned from this project may inform other art and science collaborations. The letter also includes perspectives from three Art x Climate artists and reflections on moving forward.

## Introducing art to NCA5

### Establishing a process for incorporating art into the NCA

The Art x Climate process was developed by an interagency collaboration between USGCRP, the Smithsonian Institution, the National Oceanic and Atmospheric Administration, the National Science Foundation, and the Federal Emergency Management Agency. Together, these agencies determined the timeline for the project, consulted with legal counsel to write the rules for submission, and developed the selection criteria.

The project began with two public calls for art, one for youth (ages 13–17) and one for adults (ages 18 +). The calls invited artists to contribute works that visualize climate change in the United States: its causes and impacts, vulnerabilities, and responses. The calls were communicated through the USGCRP newsletter and social media channels, highlighted on challenge.gov and a White House blog post (Crimmins 2022), and posted to several freely accessible call-for-art websites. Art x Climate attracted widespread interest, with more than 800 submissions from across the country. The submissions were received electronically, and artists were asked to provide a brief written statement.

Artwork that met the published criteria were transmitted to a jury of experts at the art–environment–science nexus. Jurors came from institutions such as the School of the Art Institute of Chicago, the Center for Art and Environment at the Nevada Museum of Art, the National Portrait Gallery, the National Science Foundation, the New York City Climate Museum, and others. All selection steps were conducted blindly: Jurors scored art based on the established criteria without knowing the artists’ names or backgrounds and without knowing the scores of the other jurors. Based on those initial scores, the jurors convened to discuss a subset of the top artworks and further narrow the selections to build a cohesive collection.

The final collection includes 92 artworks, which are featured in an online gallery on the NCA5 website. From the collection, the jury also selected three youth award winners and two adult award winners, who were featured in a White House press release (The White House 2023c) and received modest monetary prizes. Artworks were used in the banners preceding chapters and embedded within chapter narratives, where specific design elements are used to distinguish artworks from scientific figures.

### Insights for success

The success of the Art x Climate project (see Sect. 4 below) can likely be attributed to a combination of factors:

- **Supportive leadership** was critical, especially when championing the project among scientific groups for whom art–science collaboration is novel. Leadership support helped to clear potential obstacles, access funding, and attract collaborators.
- **Resources** included approximately $5,000, which supported the use of an art submission and evaluation platform, as well as modest awards. Dedicated staff time, particularly by someone with experience working in the arts, allowed for effective project execution.
- **Community input** from individuals and programs with experience in the art–climate–science arena was formative. Art x Climate leads spoke with experts from the Cultural Programs of the National Academy of Sciences, the National Endowment of the Arts, and the Portland Museum of Art, whose Tidal Shift project served as inspiration. These conversations helped frame project goals.
- **A thorough process** provided a guiding framework for Art x Climate. For example, the call included a formal legal agreement with stipulations around issues such as copyright, intellectual property, likeness permissions, and privacy rights. Separately, the jury process was carefully designed to limit bias. Such processes were designed to protect participants and grant legitimacy to the project.
- **The importance of climate change** and the **high profile of the NCA** also likely contributed to the success of Art x Climate. Climate change is a topic of growing prominence in the national discourse. The NCA is the United States’ preeminent report on climate change—the fourth assessment received over 1 million views around the world. This combination likely inspired the volume of submissions, despite the fact that Art x Climate was a first-of-its-kind effort.

## Spotlight on three Art x Climate artists

This section highlights the work of Art x Climate artists Ian van Coller, Laura Tanner, and Michael Snyder. Their work reflects the breadth of NCA itself, speaking to the physical climate system, the impacts of climate change, and the ways in which people are responding. Their works also evoke many of the same principles involved in developing climate assessments (Crimmins et al. *in this issue*) (n.d.), for example, co-production, cross-disciplinary collaboration, and connecting climate change with justice.

### Ian van Coller: co-producing art with scientists

**Fig. 1:**
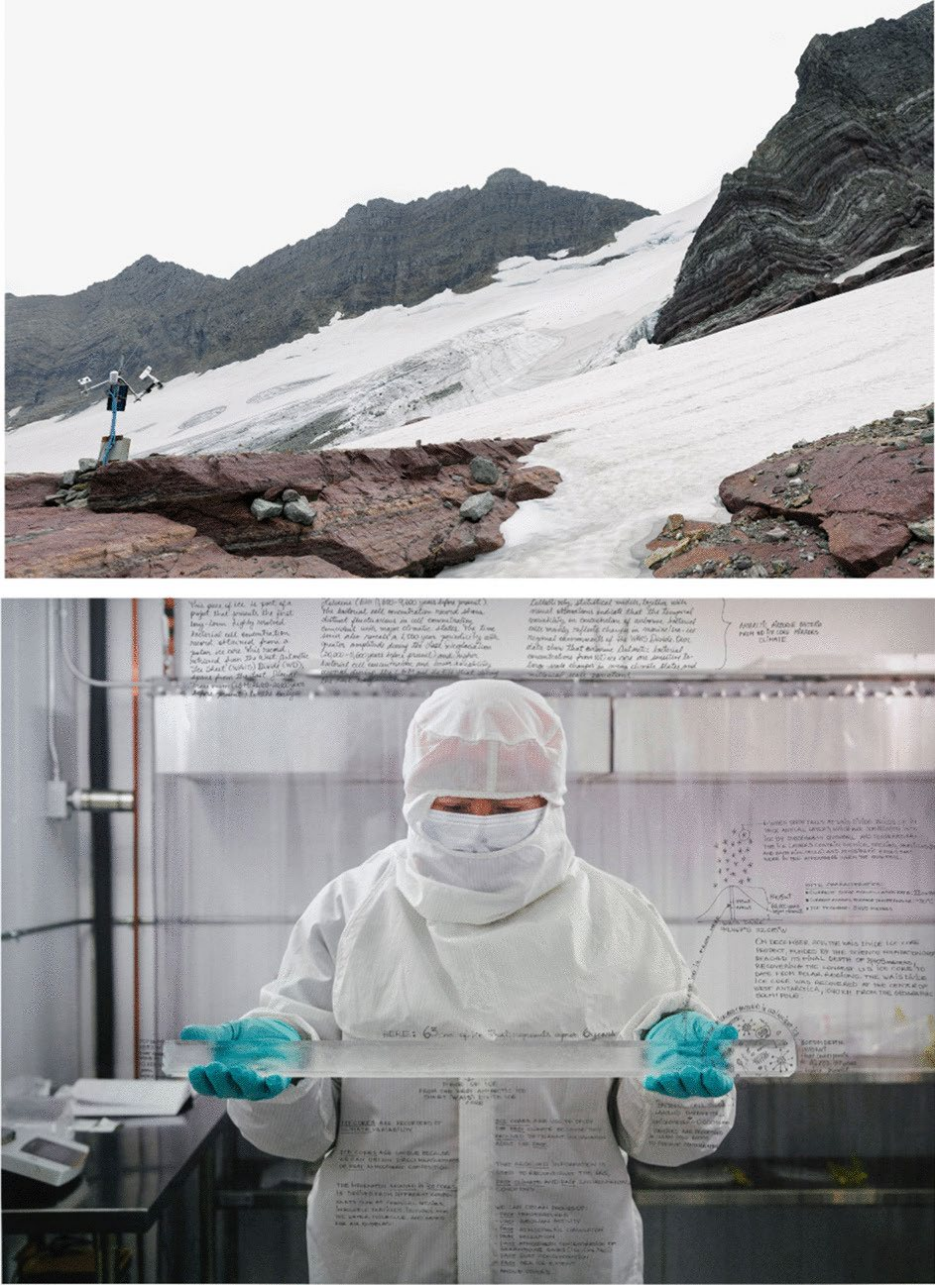
(top) Sperry Glacier weather station in Glacier National Park, where van Coller began to think about working with scientists. In his work he aims to make science more accessible to a larger audience and allow scientists to explore their own creativity. (bottom) *Dr. Avila Holding Cut Antarctic Ice Core*, 2017. Dr. Avila is holding a piece of an ice core—the left side is from 10,827.43 years before present and the right side is from 10,833.49 years before present. The core was retrieved from a depth of 1,884 m at the West Antarctic Ice Sheet Divide in 2011

Climate change has compressed and conflated human and geologic time scales, making it essential to find ways to conceptualize “deep time.” Ian van Coller’s project, *Naturalists of the Long Now*, seeks to make notions of deep time comprehensible through visual exploration of glacier ice, as well as other earthly archives. Initially inspired by the 10,000 Year Clock Project of the Long Now Foundation, van Coller collaborates with paleoclimatologists to make art that challenges viewers to think about the vast scales of geologic time that are recorded in the Earth’s ice bodies, trees, sediments, and fossils. Van Coller photographs the sites where scientists conduct their research and then asks that researcher to interpret the scene by annotating directly onto a large photographic print (Fig. 1).

In the piece *Dr. Avila Holding Cut Antarctic Ice Core*, van Coller photographed Dr. Pamela Santibáñez-Avila through a glass window in a level one clean lab on the campus of Montana State University. The annotations by Dr. Avila are carefully didactic, expressing the importance of ice-core research and what it can tell us about Earth’s past atmosphere and climate. As Dr. Avila writes in her annotations, “that archived information is used to reconstruct the past, past climate and past environmental conditions.”

### Laura Tanner: connecting climate change and culture

**Fig. 2:**
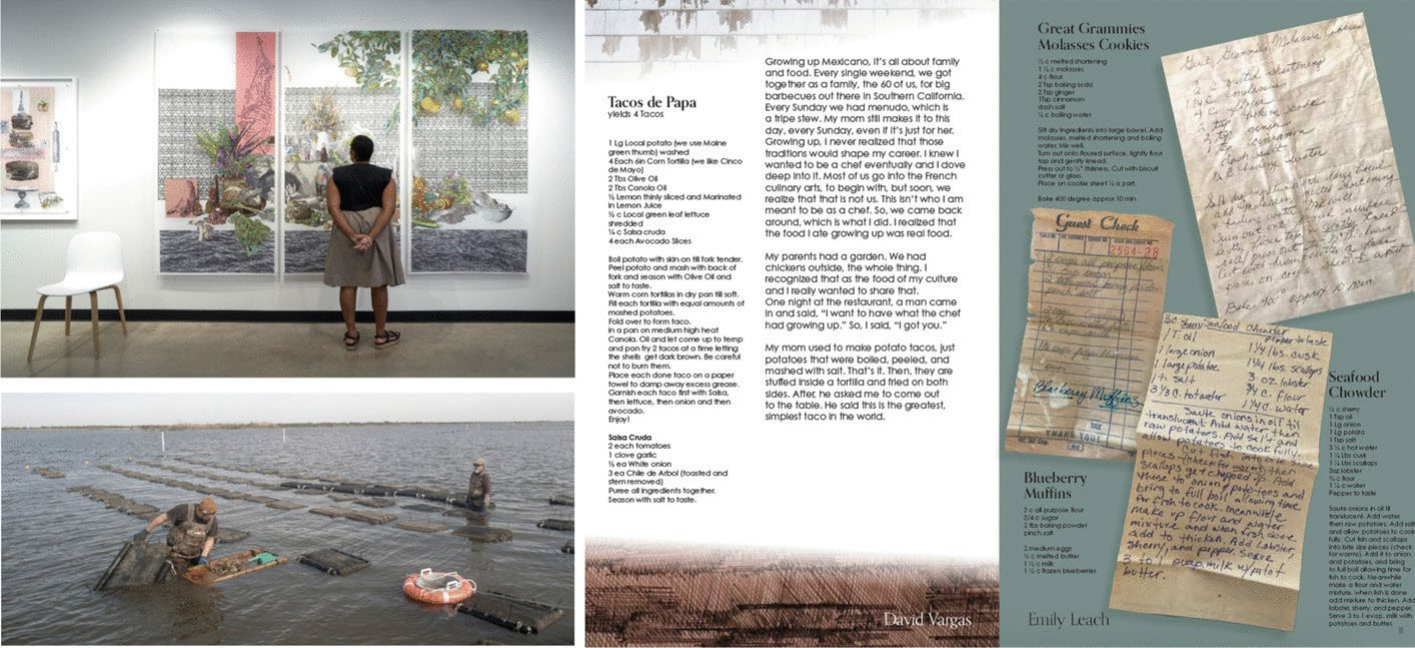
(top left) Installation detail in Tucson, AZ. *Dish,* ink and gouache on hand-cut mylar, 2022. (bottom left) Justin and Terry Trosclair tending to their oyster farm in Grand Isle, LA, which has been impacted by repeated hurricanes. Their stories about the challenges of maintaining their farm are documented in the catalog, *Dish (Waterways): Stories from the coasts of New England, Southeast Louisiana, and South Florida.* (right) Pages from *Dish (Waterways)*. The stories included here are inspired by residents living in coastal communities that rely heavily on tourism and fishing—industries under threat due to saltwater intrusion into local estuaries

Laura Tanner’s current project, *Dish*, employs interdisciplinary forms of storytelling—visual, written, and oral—to explore the intersections of climate and social justice. In 2021, Tanner began approaching these subjects through the study of foodways. To do this, she organizes community meals and recipe exchanges whereby local residents, food providers, and scholars can share stories and food traditions related to the gathering table. Tanner archives the recipes, stories, and photographic documentation of the event in a series of drawings, short films, and a printed catalog (Fig. 2). The catalogs take the form of a community/Junior League cookbook, acknowledging the inherent history of activism embedded within informal documents typically produced within the domicile (Edge 2018).

Research for *Dish* engages with scholars across disciplines, including anthropology, sociology, and the environmental sciences, incorporating various research methodologies into the creative process. Tanner spends extended periods of time in the communities referenced within her work, engaging with local residents and food providers; accompanying them through their daily routines; and discussing how the landscape, food systems, and public policies have changed during their lifetime. The translation of these oral histories into drawings and other creative media expands the conversation beyond the academic community and makes current research more accessible to the public.

### Michael Snyder: collaborating with communities

**Fig. 3:**
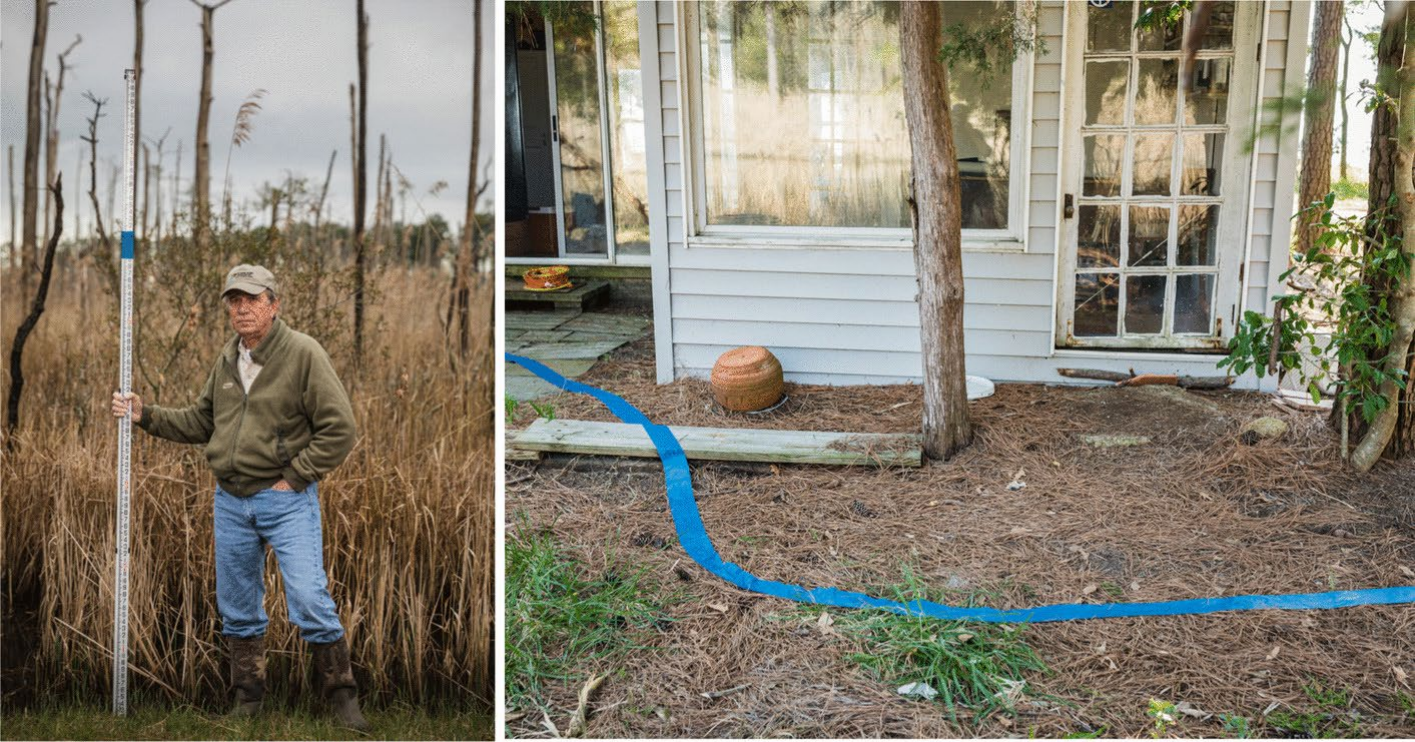
(left) Donald Webster in Dorchester County, Maryland holds a depth stick showing 6 feet of sea level rise and describes local perspectives on climate change: “A lot of people can’t stand to bring it up at all. Because, you have to understand, this is heartbreaking. Can you imagine losing your place? Folks here wouldn’t rather live anywhere else on earth … So, it is really hard for people to admit it or talk about it … But, if you can’t see it now, you never will.” (right) Blue tape runs through a yard in Royal Oak, Maryland, marking the potential future coastline

From 2006 to 2015, global sea levels have risen by 3.6 mm per year (Lindsey 2022). While this is a rapid rate of change from a geologic perspective, it’s hardly noticeable to the human eye. Michael Snyder’s project, *The Coming Coast* (Fig. 3), aims to make sea level rise visible by using creative photography and visual storytelling. For the project, Snyder traveled the 11,000-mile coastline of Chesapeake Bay of Maryland and Virginia. Using a Climate Central coastal risk screening tool, he used blue painters’ tape to mark the path of the coastline projected in the year 2100 (median projection under RCP8.5 as described in Kopp et al. 2017). Snyder also met with 31 residents from the area and photographed them holding a depth stick showing projected sea level rise at a place meaningful to them.

Snyder takes a collaborative, longitudinal approach to his work, creating projects in partnership with communities. Snyder often spends months or years building relationships and, wherever possible, focuses on uplifting individual perspectives using oral histories. His work focuses on bridging scientific data with personal, emotive stories of hope, solutions, and visions for the future, knowing that these narratives are more likely to inspire change than presenting data or disaster narratives alone. Given the importance of reciprocity in storytelling, Snyder has hosted several shows of the work in the Chesapeake region, using the events as opportunities for community dialogues guided by local leaders. Snyder has also taught his storytelling technique to local colleges and high school students, who are now creating their own versions of *The Coming Coast*.

## Lessons learned from Art x Climate

### Interdisciplinary projects require mutual respect for art and science, artists and scientists

**Fig. 4:**
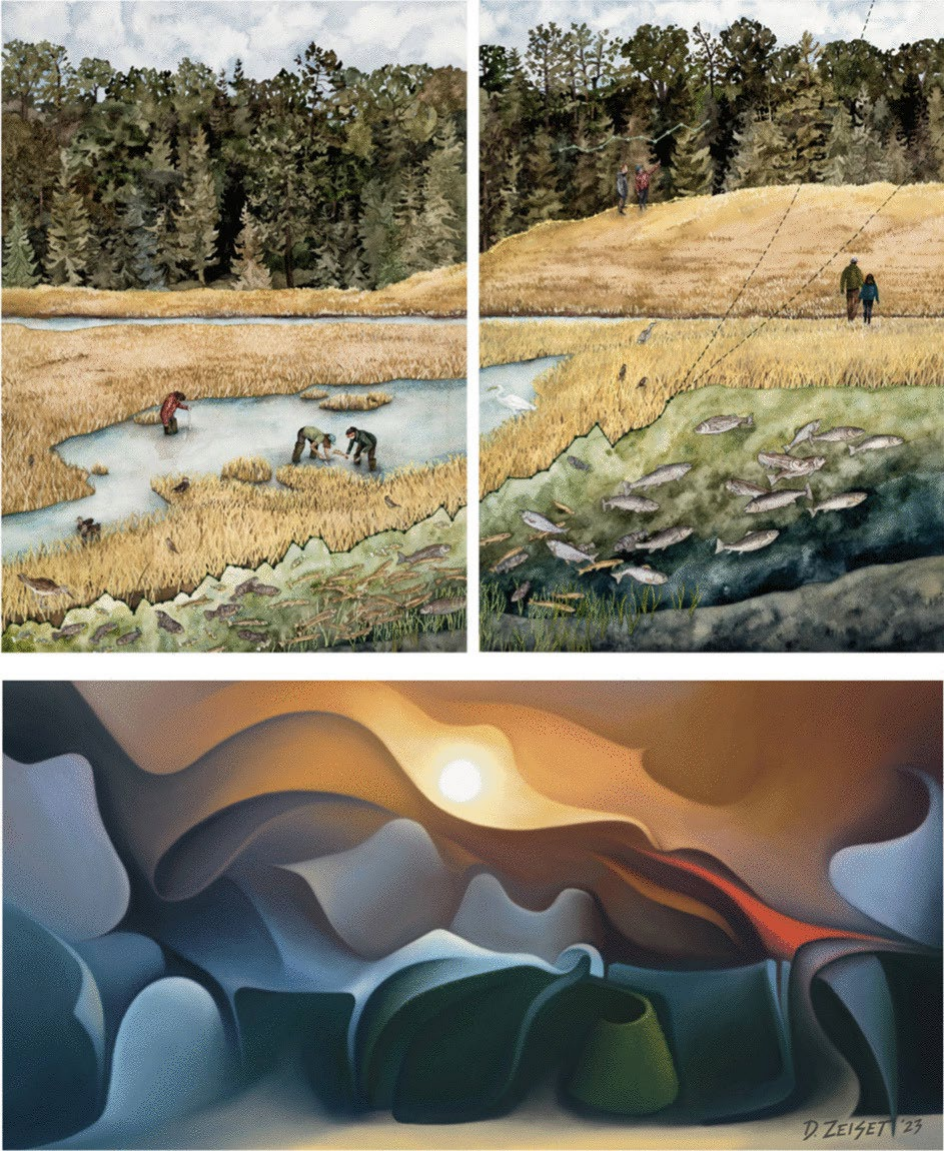
(top) *Replanting Resilience (Diptych)* by Jillian Pelto (watercolor and colored pencil, 2022). (bottom) *Firestorm’23* by David Zeiset. Two pieces from the Art x Climate collection: Pelto’s piece integrates three different datasets and uses illustrative visual storytelling in a way that is akin to science communication. Zeiset’s piece is abstract and conceptual, aiming to raise awareness of the strength and power of fire but without referencing scientific methods

Throughout the process, Art x Climate organizers have been careful to distinguish art from science communication. Especially coming from a major scientific institution like USGCRP, such framing is critical for enabling respectful cross-disciplinary collaboration. The need to make this distinction became clear at the outset of the project, as it was fairly common for scientists to think of art as illustrative or as a tool for data visualization—in a sense, art *in service* of science. While some art does include or take inspiration from data or science (Fig. 4), in many cases art is its own way of observing, imagining, documenting, interpreting, and communicating. The purpose of Art x Climate was not to better explain the scientific text of the chapters but to elevate art as a distinct way of knowing about climate change.

Many NCA5 authors appreciated the art used throughout the assessment. Some expressed desire to work with artists more outside of the NCA and noted that they had not done so because they felt intimidated given their lack of artistic expertise. Conversely, several Art x Climate artists expressed similar feelings about reaching out to scientists. Projects like Art x Climate can serve as a bridge between these two communities, dispelling the myth that artists or scientists are unapproachable. The high level of interest from both scientists and artists suggests an opportunity to further catalyze collaboration in the future. Such collaboration could also help to address the long-standing gap in climate-related funding for the arts (Overland and Sovacool 2020).

### Climate change art takes many forms

The call for art for NCA5 was restricted to visual mediums. The interagency planning committee made this decision in part to limit the scope of the project, as it was the first time USGCRP had attempted this type of call for input. Visual media also allowed for easier collection of electronic submissions and could be incorporated into the existing website development requirements for the final NCA5 report.

Even with this restriction, there was a diversity of artworks submitted. The Art x Climate collection includes professionals and amateurs across a range of mediums such as painting, drawing, textile, collage, performance, photography, sculpture, and installation. USCGRP received feedback expressing interest in other forms of art for future calls, including music, poetry, videos, and dance.

The submissions were also diverse geographically, with all 10 NCA regions represented (Avery et al. 2023), and thematically. Themes include, for example, wildfire, glaciers, extreme events, uncertainty, and resilient futures. Many pieces within the Art x Climate collection focus on impacts and lived experiences, conveying grief, anxiety, and loss. Other artworks speak to human connections with the natural world and the potential to respond to the climate crisis. This thematic diversity in submissions may be due to the fact that the NCA itself covered a very broad range of topics. Furthermore, climate change is no longer a future abstraction; the impacts of climate change, particularly extreme events, are being felt by people around the country, across physical and social geographies (Jay et al. 2023).

The large number of submissions could be attributed to the fact that the NCA is a widely recognized and authoritative resource on climate change with hundreds of thousands of readers worldwide. However, most of the submissions were in the adult category, with fewer than 100 entries in the youth category. The smaller number of submissions by youth could be attributed to the minimum age requirement (13) or that the call for art did not sufficiently reach younger audiences. Youth participation could be improved by using other communication methods; broadening outreach; and, on a larger scale, by reinvesting in youth art curricula in primary schools.

### The arts expand the reach of the climate conversation

Since the publication of NCA5, museums and science organizations across the country have reached out with keen interest in Art x Climate. USGCRP has collaborated with the San Francisco Exploratorium, the New York City Climate Museum, the Smithsonian’s National Museum of Natural History, and others to put on public programming and exhibits featuring NCA scientists and artists in conversation. The Association of Science and Technology Centers developed an NCA5-focused training program, which includes a special section Art x Climate, for over 60 museums. The Association suggests using the collection for professional development and youth engagement, noting that artwork makes clear that there “aren’t necessarily black and white answers to questions about how our society should respond to climate change—just as artworks show a range of perspectives… so do societal responses to climate change” (ASTC 2024). The NCA5 art is also featured in the updated Climate Literacy Guide, which provides an educational and communication framework of principles and concepts (USGCRP 2024).

Art x Climate has seen similar success outside of institutional collaboration. The effort has been recognized at the highest levels of government, with President Biden spotlighting the project in his Arts and Humanities Month proclamation and inviting one of the youth artists to introduce him at the NCA5 release announcement (The White House 2023b). The Art x Climate gallery is the second-highest-viewed page of the NCA5 website, following the Overview chapter. Other science programs and assessment managers, within the U.S. and abroad, have inquired about lessons learned as they consider similar projects. The project has been featured by national media, local news, and academic outlets. There are likely additional outcomes of the Art x Climate effort that can never be fully known or quantified, for example, helping people to see themselves in the report and feel welcome in the climate conversation more broadly, allowing people to connect to the issue in ways that they previously had not, making the topic of climate change more approachable or engaging, or inspiring future career choices.

## Concluding thoughts

The NCA makes clear that climate change is occurring in the present and that future risk hinges on action in the present (Jay et al. 2023). Across the country, innovations are emerging, communities are responding, and we are beginning to transition towards a more sustainable world. At the same time, additional, transformative change is required to address the challenges posed by current and future climate change (Wasley et al. 2023). Like climate change itself, the way we respond to the climate crisis can be difficult to imagine, as the need for transformational change challenges our ability to envision a radically different future.

Art provides inspiration for, and is a mode of, such transformational change. Art is a way of knowing and a way of storytelling—the stories told today have the potential to give rise to action for the future (Sommer and Klöckner 2021). Art x Climate demonstrates the many ways that the arts convey the realities of climate change: by processing the past and the systemic conditions that gave rise to the challenges of today; by documenting consequences of extreme events and other impacts; and by envisioning the future and centering stories of hope, leadership, and innovative climate solutions.

Telling these stories means creating space for the arts to be included in the climate change conversation and in dialogue with science. For artists it means centering climate change as one of today’s most pressing issues and featuring the topic more prominently in artistic communities. For scientists it means recognizing the arts as a substantive part of understanding and response. For example, climate assessment developers might consider hosting participatory art programming during engagement events to gather data on lived experience, to support dialogue between scientists and assessment users, and to encourage more participation by youth (Bentz 2020). Assessments could similarly provide a launchpad for a collaborative art-science residency that endures over the course of the assessment cycle. Future assessments could also include multimedia storytelling, particularly to highlight local community actions. Interdisciplinary experts who can bridge these spaces are critical to facilitating the kinds of collaborations needed to tackle the climate crisis.

## Data Availability

No data was used in this manuscript. The full Art x Climate gallery can be found at nca2023.globalchange.gov.
